# The Role of Bacterial Membrane Vesicles in Human Health and Disease

**DOI:** 10.3389/fmicb.2022.828704

**Published:** 2022-03-01

**Authors:** Daniel N. Villageliu, Derrick R. Samuelson

**Affiliations:** Division of Pulmonary, Critical Care, and Sleep, Department of Internal Medicine, University of Nebraska Medical Center, Omaha, NE, United States

**Keywords:** bacterial membrane vesicles, microbial endocrinology, OMV, bacterial nanoparticles, immunity, signaling

## Abstract

Bacterial membrane vesicles (MVs) are nanoparticles derived from the membrane components of bacteria that transport microbial derived substances. MVs are ubiquitous across a variety of terrestrial and marine environments and vary widely in their composition and function. Membrane vesicle functional diversity is staggering: MVs facilitate intercellular communication by delivering quorum signals, genetic information, and small molecules active against a variety of receptors. MVs can deliver destructive virulence factors, alter the composition of the microbiota, take part in the formation of biofilms, assist in the uptake of nutrients, and serve as a chemical waste removal system for bacteria. MVs also facilitate host–microbe interactions including communication. Released in mass, MVs overwhelm the host immune system and injure host tissues; however, there is also evidence that vesicles may take part in processes which promote host health. This review will examine the ascribed functions of MVs within the context of human health and disease.

## Introduction

Bacterial membrane vesicles (MVs) vary in size from 20 to 500 nm and are surrounded by a membrane bilayer derived from bacterial lipids and proteins (Bos et al., 2021). Shielded by this membrane, the lumen of a MV is a protected microenvironment which allows for the transport of biological molecules that would otherwise be inactivated or diluted to inefficacy by environmental exposure (Kadurugamuwa and Beveridge, 1995). MVs have been observed for all Gram-negative bacterial species studied to date (Gao and van der Veen, 2020), as well as Gram-positive bacteria, including *Staphylococcus aureus*, *Enterococcus faecium*, *Clostridium perfringens*, *Mycobacterium ulcerans*, *Bacillus* spp., and *Lactobacillius* spp. among other accumulating examples (Dorward and Garon, 1990; Marsollier et al., 2007; Kim et al., 2009; Lee et al., 2009; Dean et al., 2019).

**Figure 1:**
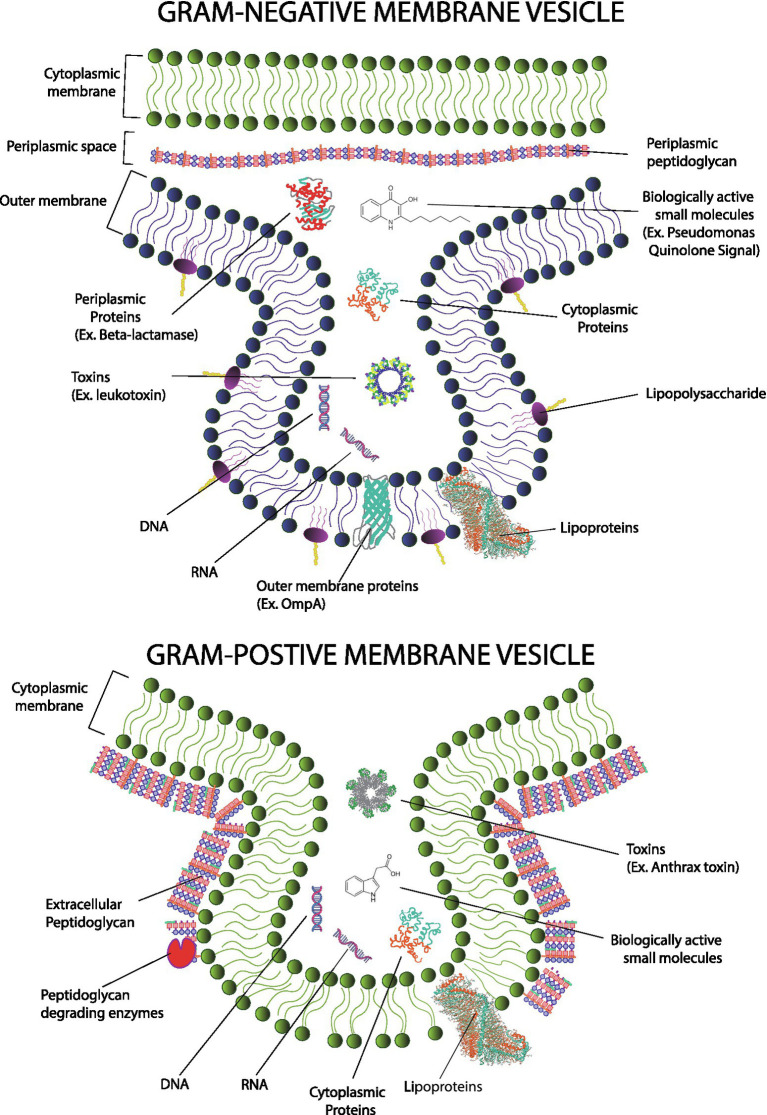
Gram-positive membrane vesicles (MVs; top) form from the cytoplasmic membrane and encapsulate cytoplasmic material. In contrast, Gram-negative MVs (bottom) form primarily from the outer membrane and encapsulate material from the periplasmic compartment; however, contributions from the cytoplasm and cytoplasmic membrane have been reported in Gram-negative species. Mechanisms of MV formation differ and mechanisms of Gram-positive MV formation must overcome an outer layer of peptidoglycan. Gram-negative MVs and Gram-positive MVs carry similar cargos including genetic information, proteins (including toxins and enzymes) as well as small molecule messengers. However, because of evolutionary divergence, specific types of molecules are not shared. For example, LPS and many types of outer membrane proteins are found only in Gram-negative microbes.

Gram-negative MVs are frequently referred to as outer membrane vesicles (OMV; Lee et al., 2007), while Gram-positive MVs are referred to as cytoplasmic membrane vesicles (CMV; Vitse and Devreese, 2020). The names, though established, are somewhat misleading as we now know that variations of vesicles can exist which include components from the inner membrane, periplasm, or cytoplasm (Figure 1). For simplicity, this review will favor using the general term “MV” whenever possible.

Gram-negative MVs typically consist of material found in the Gram-negative outer membrane but can also package material from the cytoplasm and periplasm as well. They contain lipids, including lipopolysaccharides (LPS), glycolipids, and phospholipids; nucleic acids including genomic and plasmid dsDNA, small RNA; and proteins including membrane proteins, enzymes, and toxins (Klieve et al., 2005; Lee and Hong, 2012; Bitto et al., 2017).Gram-positive MVs carry many chemicals like those found in the MVs of Gram-negatives microbes with some important distinctions (Brown et al., 2015; Kim et al., 2015). MVs from a Gram-positive microorganism derive from the cytoplasmic membrane, not the Gram-negative outer membrane. Obviously, there exists a plethora of differences between these two types of membranes and those differences are reflected in the composition of their respective MVs. For example, Gram-positive MVs do not contain LPS as LPS is specific to Gram-negative bacteria (Ñahui et al., 2021). This is a noteworthy distinction because LPS is profoundly immunogenic.

One would expect that the composition of a MV would dictate the effects it can exert upon a host. A corollary to this would be that the effects of MVs derive from the factors which influence the composition of MVs. The formation and composition of MVs are affected by environmental conditions, including temperature, nutrient availability, oxidation, and the presence of triggers such as toxins or quorum sensing molecules (McMahon et al., 2012; Macdonald and Kuehn, 2013; Abe et al., 2021; Briaud et al., 2021). An important healthcare related example is that MVs formed from bacteria exposed to antibiotics have the potential to be more virulent than MVs generated in the absence of antibiotics. We will discuss this example in more depth in a later section.

Researchers exploring the composition of MVs often compare the lipid and peptide composition of a membrane vesicle to the composition of the parental cell. Experiments of this nature have given us evidence that the composition of MVs can be generated semi-selectively and regulated. Bitto et al. (2021) reported differences in western blot muropeptide banding between MVs and the whole cell lysate of parent *S. aureus*; in particular, they noted the presence of a 60 kDa protein present in MVs that was not present in cell lysates and the presence of a 37 kDa protein in cell lysates that was not observed in the MVs. The author’s suggested that these differences could have arisen from modification of peptidoglycan moieties during MV formation, a finding which hints at a mechanism by which MVs form in *S. aureus* (Bitto et al., 2021). For some bacterial species, the mechanisms of MV formation are better understood, and scientists can even generate MVs carrying specific proteins by including elements like the OmpA signal (Carvalho et al., 2019). This has proved useful in the development of MV vaccines which are engineered to express antigens capable of training the immune system.

We will now take some time to discuss known mechanisms of MV formation. Though the connection of this admittedly arcane topic to human health may at first seem tenuous, the reality is that the mechanisms of MV formation influence the composition of MVs and their probable roles. When making attempts to classify different MVs, researchers often include pathway of genesis in addition to considerations of their origin, contents, and apparent function (Nagakubo et al., 2020).

Toyofuku et al. (2019) broadly categorized the routes which form MVs. The mechanisms of formation for Gram-negative microbes include MVs that result from blebbing and MVs that result from explosive cell lysis (Toyofuku et al., 2019; Nagakubo et al., 2020). Briefly, blebbing can result from mechanisms which disrupt the cross linkage between the inner and outer membrane or from the accumulation of agents which induce positive curvature in the outer membrane. Examples of the latter include the accumulation of misfolded proteins, phospholipids, or anionic B-band LPS. When positive curvature and internal turgor pressure is sufficient, budding MVs will be pinched off from the mother cell. The formation of Gram-negative membrane vesicles *via* membrane blebbing also appears to be modulated by specific proteins and pathways. For example, a study in *Salmonella enterica* identified nine genes, which influence the biogenesis of MVs (Nevermann et al., 2019). These genes include envC (encoding a murein hydrolase activator involved in remodeling of peptidoglycan), mrcB (encodes an enzyme which influences cell wall formation through the synthesis of peptidoglycan cross-linkages), rfaE, and waaC (genes associated with LPS synthesis), ompA, nlpI, and tolR (gene products are associated with envelope fluidity), degS (stress sensor serine endopeptidase), and hns (global transcriptional regulator). Genes related to stress responsive elements are particularly interesting because they suggest possibilities by which exogenous stressors, like antibiotics, might upregulate MV production.

Explosive cell lysis can occur following damage to the peptidoglycan portion of the cell wall as may occur following antibiotic exposure, phage infection, or activation of bacterial endolysin as part of an SOS response. Without the cell wall to counteract hydrostatic pressure, mechanical stress fragments the membrane explosively. Fragmented membrane may then reform smaller spherical particles through hydrophobic interaction. Presumably, membrane vesicles formed in this manner would be unlikely to carry selective contents. However, MVs formed in this manner are thought to be important to the formation of some biofilms. Turnbull et al. (2016) documented that explosive cell lysis in *Pseudomonas aeruginosa* liberated cytosolic proteins, eDNA and MVs that contributed to the formation of biofilms in *P. aeruginosa*. They also determined that explosive cell lysis in *P. aeruginosa* was dependent on an endolysin-encoding gene (lys) located in a genetic region known to be upregulated by exogeneous stresses through the RecA-dependent SOS response (Turnbull et al., 2016).

In contrast to work on Gram-negative microbes, the study of MV formation in Gram-positive microbes is relatively nascent. As far back as 1973, there were researchers who claimed that membrane vesicles could be isolated from Gram-positive *Bacilli* including *Bacillus subtilis*; some of this was even substantiated by transmission electron microscopic analysis of material purified from *B. subtilis* in 1990 (Konings et al., 1973; Dorward and Garon, 1990). However, until recently, the larger scientific community dismissed these reports of MV formation in Gram-positive microbes. In part this may be due to erroneous assumptions on the nature of the Gram-positive cell wall. We now recognize that the Gram-positive cell wall exists in a managed homeostatic equilibrium which constantly balances synthesis and degradation (Atilano et al., 2010; Hanson and Neely, 2012). In 2009, Lee et al. (2009) described MVs from *S. aureus*; more recently, several distinct mechanisms have been described which act on the cytoplasmic membrane and peptidoglycan layers to facilitate MV production in *S. aureus*. At the cytoplasmic membrane level, amphipathic molecules called phenol-soluble modulins increase membrane fluidity; the increased fluidity allows membrane budding *via* osmotic forces (Hanzelmann et al., 2016; Schlatterer et al., 2018). At the cell wall, porosity is modulated by the production of autolysins and agents which alter peptidoglycan cross-linking (Wang et al., 2018). As pores open in the thick peptidoglycan layer, underlying buds can now be secreted by bubbling through the pores.

What is the fate of MVs after they are secreted? Secreted membrane vesicles often remain associated with their host cell. These vesicles form concentric rings around the producing microbe or aggregate themselves into larger structures, which bridge adjacent cells (Bos et al., 2021). MVs distributed in this manner serve as a type of armor, offering up sacrificial membrane that improves resistance against phage or environmental insults like antibiotics which would otherwise directly damage the envelope of the mother cell (Manning and Kuehn, 2011; Sabnis et al., 2018). Enzymes, including β-lactamases, which are contained in this “MV armor” can attenuate the effects of antibiotics (Lee et al., 2013). Some species, such as Gram-positive *Streptococcus suis*, have also been reported to produce MVs with enzymes that actively degrade host defense antibodies (Spoerry et al., 2016).

In contrast to membrane vesicles which remain associated with their producing cell, some MVs migrate and diffuse throughout extracellular space. Crucially, MVs which diffuse away from their producing cell can precipitate distant changes. This “action at a distance” is particularly relevant for MVs carrying cargos such as toxins and later, we will discuss examples of microbe-host interaction facilitated by MVs that have travelled systemically.

For this review, we will begin with a discussion of host-MV interaction at a general level, examining the health impact from environmental exposure to MV, as can occur with MV found in airborne dust or in food. This will be followed with sections detailing specific MV activities that hold relevance to pathogenesis or other health considerations. Sections which discuss the virulent potential of MVs are compulsory; however, it is also worth examining the evidence for how MVs can participate in host-microbe signaling or even promote health.

## Health Impact From Exposure to Environmental Membrane Vesicles

Durable MVs persist and accumulate within the environment; this is evidenced by the fact that MVs are ubiquitous across marine and terrestrial environments. In these environments, MVs are thought to assist bacteria in overcoming environmental challenges. For example, MVs exist which catalyze complex biochemical reactions like the digestion of lignin derivatives in the soil (Salvachúa et al., 2020); in marine environments, there are MVs which appear to mediate energy and nutrient transfers (Biller et al., 2021). One common source of exposure to environmental MVs is organic dust (Meganathan et al., 2020). However, what are the effects of these “environmental MVs” on host-health? We now have some insight into the effects of aerosolized MVs, such as might be encountered in dust.

It has been well-documented that respiratory exposure to ultrafine particles (<100 nm) can increase the risk of pulmonary disease. Submicron particles penetrate deeply into pulmonary tissue and reach the thin non-ciliated surfaces of the alveoli (Thomas, 2013). Further, total particle deposition increases for particles smaller than 100 nm (Heyder, 2004). MVs are unique among submicron particles as they not only have the physical characteristics necessary for deep respiratory penetration, but they also can contain adhesive surface proteins, and immunogenic features like LPS. LPS is particularly noteworthy because even relatively low levels of inhaled LPS can trigger an immune cascade leading to Th2 cell induction and subsequent IL-5 and IL-13 release. The release of these interleukins is thought to be central to the pathogenesis of asthma (Yang et al., 2020). Though LPS is an important component of Gram-negative MVs, other vesicular components should not be neglected. Isolated gram-negative vesicles can elicit a stronger pro-inflammatory immune response than purified LPS (Ellis et al., 2010). Gram positive MVs, devoid of LPS, can also elicit potent immune responses, both pro-and anti-inflammatory (Yang et al., 2017). Results such as this highlight the importance of understanding the composition of membrane vesicles and how they can vary.

Though less studied, there is evidence that ingested MVs might have the potential to influence host health. In a study of the uptake, trafficking, and bio distribution of MVs generated from *Bacteroides thetaiotaomicron*, Jones et al. (2020) demonstrated that MVs administered orally can reach systemic tissues. In a murine model, *B. thetaiotaomicron* MVs were labelled with a far-red fluorescent dye (DiD). Labeled MVs were orally administered 8 h prior to organ excision and subsequent fluorescent imaging. Tissue from MV treated animals demonstrated an increase in far-red fluorescence. The highest level of increase was demonstrated for tissue of the gastrointestinal tract, particularly the small intestine. Strong signals were also observed in the stomach, caecum, and colon. Systemic tissues including the liver, lungs, and heart also demonstrated increases; for these tissues, the effect was most pronounced for the liver. This data demonstrate the capacity for MVs to diffuse from the gastrointestinal tract to reach distal host tissues. The observation that MVs can achieve systemic distribution following gastric passage is also important. Consider that the bacterial contents of the gastrointestinal tract are not exclusively limited to products produced by the host microbiota.

A wide variety of fermented products are widely consumed throughout the world: Western dietary staples such as yogurt and cheese and Eastern examples including fermented vegetables, fish, and meat (Tamang et al., 2020). Probiotics are also growing in popularity. While there exists a large body of popular media which promotes the health value of fermented foods or various probiotics, in most cases, a mechanistic understanding has yet to be elucidated (Fijan, 2014). For probiotics in particular, pharmacological questions concerning optimal dosing and means of preparation have yet to be settled by scientific evidence. In some cases, it is debatable whether a probiotic has the capacity to colonize or produce salubrious effects within the gut environment. The contribution that microbial vesicles might make to the diet remains unclear. Consider however, that typical probiotic preparations do not exclude membrane vesicles, instead concentrating a variety of microbial products (Fenster et al., 2019). When administering probiotic “crumbles” or other blends of probiotics, one is in effect also dosing a crude mixture of secreted extracellular material including membrane vessels. As such, it is impossible to rule out the possibility that many probiotic benefits may derive from MVs. A similar argument can also be made for fermented foods.

Whether gastrointestinal MVs arise from the gut microbiota or ingested contents, MVs that enter the systemic circulation *via* migration through the gastrointestinal tract have the potential to exert influence on a variety of host tissues (Choi et al., 2015; Yu et al., 2018). Unfortunately, many MVs appear to have deleterious effects on the host.

## Vesicles Delivering Virulence Factors

Membrane vesicles have been widely studied for their ability to contribute to bacterial pathogenicity through their delivery of virulence factors. Vesicle carried virulence factors have now been described for many pathogens, including *P. aeruginosa*, *S. aureus*, *C. perfringens*, *Escherichia coli*, *Moraxella catarrhalis*, *Porphyromonas gingivalis*, *Helicobacter pylori*, and *Neisseria gonorrhae*. Bacterial toxins, adhesins, invasins, outer membrane proteins, lipoglycans, LPS, flagellin, and proteases have all been identified as constituents of bacterial MVs (Table 1). By delivering lytic enzymes or toxins, MVs serve as a bacterial “long distance weapons” which help the bacteria compromise host defenses and excel within their niche (Rueter and Bielaszewska, 2020).

**Table 1:** Reported examples of cargo transported by bacterial membrane vesicle.

Gram-negative species	Cargo	Purported function	References
*Acinetobacter baumannii*	Outer membrane protein A (AbOmpA), subunits of Csu pili, and immunomodulatory surface proteins	Induces cell death through mitochondrial and nuclear targetting (AbOmpA), adhesion to host cells and biofilm (Csu pili), and immunomodulation	Kwon et al., 2009; Mendez et al., 2012; Jun et al., 2013
*Bartonella henselae*	Hemin Binding Protein C	Protects bacteria against toxic levels of hemin	Roden et al., 2012
*Borrelia burgdorferi*	Surface proteins (OspA, B, D), enolase	Adherence to host cells (OspA, B,D), proteolysis through plasminogen activation (enolase)	Lähteenmäki., 2001; Toledo et al., 2012
*Escherichia coli*	Heat labile enterotoxin (LT), Shiga toxin (ST), Cytolysin A (ClyA), and β-lactamase	Cytotoxins (LT, ST, and ClyA), antibiotic resistance (β-lactamase)	Kolling and Matthews, 1999; Horstman and Kuehn, 2000; Yokoyama et al., 2000; Kuehn and Kesty, 2005; Chattopadhyay and Jaganandham, 2015
*Helicobacter pylori*	Biofilm associated peptide (BAP)	Enhanced biofilm formation (BAP)	Yonezawa et al., 2011
*Legionella pneumophilia*	Defect in organelle trafficking protein H (DotH)/Intracellular multiplication factor (IcmK), ProA1	Disruption of host cell physiological processes (DotH/IcmK)	Vincent et al., 2006; Jung et al., 2016
*Neisseria gonorrhoeae*	PorB	Anti-macrophage pore toxin which dissipates mitochondrial membrane potential and promotes apotosis	Deo et al., 2018
*Pseudomonas aeruginosa*	Alkaline phosphatase, Phospholipase C, proteases, and hydrolases	Degradative enzymes (Protease, hemolysin, and hydrolases), quorum sensing molecules (PQS)	Kadurugamuwa and Beveridge, 1995
**Gram-positive species**	**Cargo**	**Purported function**	**References**
*Bacillus anthracis*	lethal factor, edema toxin, and anthrolysin	Potent cytotoxins	Rivera et al., 2010
*Bacillus subtilis*	Unique lipid/proteomic profile still being investigated	Protective against environmental stressors including surfactants, O2 depletion, starvation and thermal shock.	Konings et al., 1973; Abe et al., 2021
*Bifidobacterium longum*	Mucin binding proteins	Enhanced colonization of gut-lining	Nishiyama et al., 2020
*Lactobaccilus rhamnosus*	Indole	Improved gut-barrier function, anti-inflammatory	Gu et al., 2021
*Lactobacillus plantarum*	Proteins, nucleic acids, small molecular cargo	Unclear, proven uptake by intestinal epithelial cells	Bajic et al., 2020
*Mycobacterium tuberculosis*	Mycobactin, lipoproteins including lipoarabinomannan (LAM)	Iron acquisition (Mycobactin), downregulation of CD4+ T cells (LAM)	Prados-Rosales et al., 2014
*Staphylococcus aureus*	Iron-binding factors, α-hemolysin, leukocidins, phenol-soluble modulins, superantigens, and enzymes	Membrane disruptive pore toxin (α-hemolysin), Anti-phagocyte toxin (leukocidin)	Wang et al., 2021
*Streptococcus pneumonia*	pneumolysin	Lytic pore toxin	Olaya et al., 2014

A well-studied example of MVs behaving in this manner occurs in *P. aeruginosa*. *Pseudomonas aeruginosa* produces a variety of hydrolytic enzymes, including protease, alkaline phosphatase, phospholipase C, and peptidoglycan hydrolase (Kadurugamuwa and Beveridge, 1995). Additionally, *P. aeruginosa* generates several variations of LPS, including an anionic variant (B-band LPS). Together these traits allow *P. aeruginosa* to produce highly effective and virulent MVs (Kadurugamuwa and Beveridge, 1997).

Membrane vesicles from *P. aeruginosa* consist predominantly of anionic B-band LPS, a molecule consisting of trimeric units of N-acetylfucosamine linked to manno-uronic acid. When molecules such as B-band LPS accumulate in the outer membrane of a bacterium, the localized accumulation of negative charge exerts are pulsive force which facilitates membrane blebbing and MV formation (Kadurugamuwa and Beveridge, 1995; Mozaheb and Mingeot-Leclercq, 2020). Therefore, B-band LPS can be viewed as an evolved trait which is uniquely suited to prolific MV formation. The anionic character of these MVs is also important because it allows these particles to interact with many types of cells, including bacteria and animal cells which express receptors that bind anionic sugars.

Following binding, MV entry into a target host cell can occur through a variety of entry routes, including membrane fusion, macropinocytosis, clathrin mediated endocytosis, caveolin mediated endocytosis, and non-caveolin/non clathrin mediated endocytosis (Ellis and Kuehn, 2010; Kulp and Kuehn, 2010; O'Donoghue and Krachler, 2016). In the case of *P. aeruginosa*, this facilitates the delivery of hydrolytic enzymes which degrade intercellular proteins (Kadurugamuwa and Beveridge, 1996). If taken into a cell such as a macrophage, these MVs can trigger apoptosis and the release of IL-1β (Deo et al., 2020).

In addition to the usage of hydrolytic enzymes, some bacterial species also produce vesicles which contain pore forming toxins. *Neisseria gonorrhoeae* secretes MVs enriched with the outer membrane protein PorB. Within bacteria, PorB is a voltage gated ß-barrel pore that facilitates ion exchange and the uptake of small nutrients essential for bacterial viability (Chen and Seifert, 2014). However, previous electrophysiological work has shown that PorB dissipates the membrane potential (ΔΨm) of eukaryotic mitochondrial membranes and sensitizes host cells to apoptosis (Kozjak et al., 2009). In a study conducted by Chow et al., Bone marrow-derived macrophages were treated with PorB laden MVs. Macrophage health was followed by time lapse imaging using a combination of confocal and direct stochastic optical reconstruction microscopy. One finding was that PorB embedded into the mitochondrial membrane (stained with Tom20) by 12 h post treatment. Taken together with other findings presented in the publication, Chow et al. demonstrated that mammalian macrophages uptake MVs laden with PorB. PorB internalized in this manner is recognized by the translocase of the outer mitochondrial membrane and trafficked into the mitochondria. Here it dissipates the mitochondrial proton gradient required for host ATP (adenosine triphosphate) production and helps to trigger an apoptotic cascade (Deo et al., 2018).

Gram-positive organisms also utilize membrane vesicles to carry and deliver virulent cargos. Two instructive examples include *S. aureus* and *C. perfringens*. *Staphylococcus aureus* MVs contain numerous virulence factors and other proteins relevant to pathogenesis (Lee et al., 2009; Lee, 2012). Some notable examples include: Staphylococcal protein A (SpA; An IgG-binding protein which binds IgG Fc fragments, interferes with IgG hexamer formation and blocks complement activation); penicillin-binding protein 2 and β-lactamase (can contribute to antibiotic resistance by sequestering and inactivating penicillin); ferritin and lipoprotein for high-affinity iron ion transport (promote acquisition of iron, even in the iron-restricted environment of host tissues); tissue destructive enzymes including proteolysin (allows the digestion of host proteins such as structural collagen which allows the microbe to penetrate host tissues while acquiring nutrients); and coagulase (activates host clotting pathways through the non-proteolytic activation of prothrombin; clotting appears crucial to the formation of abscesses; Cheng et al., 2010). MVs from *S. aureus* also carry a variety of immunogenic PAMPs which can bind host pattern recognition receptors and elicit potent immune responses which contribute to inflammation and disease. For example, Hong et al. (2011) demonstrated that Staphylococcal MVs contribute to the development of atopic dermatitis. Briefly, the cutaneous barrier of 6-week-old mice was disrupted by repeated stripping of surgical tape. To this stripped area, gauze soaked in MV containing phosphate buffered saline was secured using a bio-occlusive tape. MVs were reapplied on a triweekly basis before inflammation and immune dysfunction were evaluated. The authors determined that the application of MVs caused epidermal thickening with dermal infiltration by mast cells and eosinophiles. These changes were also accompanied by increased levels of IL-4, IL-5, IFN-γ, and IL-17. Hong et al. (2011) also performed *in vitro* experiments dermal fibroblasts and found that application of MVs purified from *S. aureus* ATCC 14458 increased the production of inflammatory cytokines IL-6 and macrophage inflammatory protein (MIP-1α) as well as eotaxin and thymic stromal lymphopoietin.

The Gram-positive genus *Clostridium* includes notable toxin producers including *C. botulinum*, which is famous for food poisoning; *C. tetani*, famous for the potent neurotoxin tetanospasmin; and *C. perfringens* whose alpha-toxin is well-known for its necrotizing effects in gaseous gangrene. Unsurprisingly, MVs from these potent human pathogens also can exert virulent effects. For example, the Gram-positive microbe *C. perfringens* produces membrane vesicles which dramatically upregulate the production of tumor necrosis factor alpha (TNF-α; Jiang et al., 2014). The effects of TNF-α on host tissues, including the vascular system and the role of TNF-α in septic shock are well studied. In localized peripheral tissues, TNF-α impair endothelium-dependent vasodilation which increases tissue resistance and decreases tissue perfusion (Chia et al., 2003). TNF-α has other systemic effects as well which favor a decreased cardiac output and overall drop in blood pressure (Mitaka et al., 1994). These changes can be either adaptive or dysfunctional, depending on the degree of response. In the context of septic shock, where these responses can be taken to an extreme, hemodynamic instability increases patient mortality (Cohen and Abraham, 1999; Lendak et al., 2018). As TNF-α levels rise during a *Clostridium* infection, TNF-α related inflammation and hemodynamic instability would also be expected to increase. Hypotension, high tissue resistance and low systemic resistance reduce overall tissue perfusion. A reduction in tissue perfusion in turn lowers overall tissue oxygen content and impairs immune function. From the perspective of the anaerobe *C. perfrigens*, this is quite advantageous.

In addition to delivering cellular toxins which destroy a target cell, some MVs contain virulence factors which more subtly modulate their targets. Recently, it was discovered that MVs from *Borrelia burgdorferi* counteract host superoxide production in a manner which primes the host tissue for subsequent infiltration (Wawrzeniak et al., 2020). In work conducted by Wawrzeniak et al. (2020), the neuroblastoma cell line BE2C was exposed to MVs isolated from cultures of *B. burgdorferi*. Following exposure, cellular superoxide levels were reduced in as little as 30 min. This finding is important because superoxide is part of several mechanisms of host-defense, including the oxidative burst, which rely on releasing reactive oxygen species to counteract bacterial invasion. The exact reason for superoxide reduction was not conclusively shown, however, the authors conceived of the possibility that MVs delivered enzymes which degraded host superoxide. Another possibility, unproven in *B. burgdorferi*, is that the MVs carry bacterially derived small RNAs which modulate host gene expression on a translational level. We will examine modulation of the immune system further in our section on inter-kingdom signaling.

## Membrane Vesicles as an Adaptation to Environmental Insults, Implications for Antibiotic Usage

**Figure 2:**
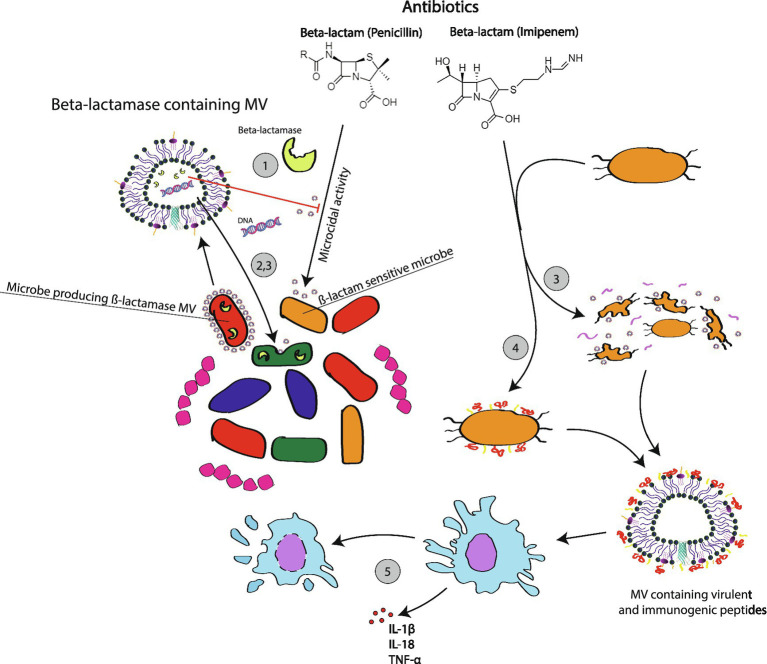
Membrane vesicle mediated microbial responses to antibiotic usage. Following exposure to antibiotics, such as the ß-lactam examples used for this figure, many microbes will be induced to activate resistant traits including antibiotic degrading enzymes. MVs made by these bacteria can interfere with the effectiveness of antibiotics through several mechamisms: ß-lactamase containing MVs can directly degrade antibiotics in the surrounding environment, which will protect the host cell and susceptible neighboring cells; (1) ß-lactamase MVs uptaken by related microbial species will confer temporary protection for cells that now carrying the ß-lactamase enzyme without the ß-lactamase gene (2); MVs can carry plasmids encoding for ß-lactamase. Plasmids horizontally transferred to other cells will impart long term genetic immunity in recipient cells as they now produce their own ß-lactamase enzymes. (3) Protective benefits do not always depend on the presence of a protective enzyme. In some cases, antibiotic exposure triggers SOS responses, which result in MV formation through routes like bubbling cell death. This appears to assist bacteria in removing disruptive waste like cellular material damaged by antibiotics. The accumulation of MVs can also contribute to the formation of a biofilm, which can non-specifically shield persister cells and limit the diffusion of antibiotics (4). There are reports of antibiotics triggering the release of virulent MVs (5). In *Klebsiella*, MVs generated following exposure to imipenem appear to trigger phagocyte pyroptosis and the release of inflammatory mediators (5).

At a time when antibiotic resistance is of growing concern, an appreciation of the contribution that MVs play in antibiotic tolerance is useful. Exposure to antibiotics can increase the production of MVs. For example, Kadurugamuwa and Beveridge (1996) reported that gentamycin use increases the production of MV in *P. aeruginosa*. This appears to be true for other species like *S. aureus* as well reference as well (Andreoni et al., 2019). Why do bacteria invest in making MVs during a time of antibiotic stress? There are several pathways by which MVs can dampen the effects of antibiotics (Figure 2).

Earlier we mentioned that MVs can carry enzymes such β-lactamases which are capable of actively degrading antibiotics. When actively secreted, these MVs provide temporary antibiotic resistance for not just the producing cell, but also other cells in a surrounding region (Stentz et al., 2015). If this region contains a complex polymicrobial community, then the presence of an organism with β-lactamases MVs will also protect surrounding species including pathogens.

Even if MVs do not carry an antibiotic degrading enzyme, MV formation may protect bacteria from antibiotics. McBroom and Kuehn tested the effects of alcohol and polymyxin B and on hyper or hypo vesiculating *E. coli* mutants (McBroom and Kuehn, 2007). Hypervesiculating mutations appeared protective; in one case increasing 2 h survival from <1% to approximately 70%. Hypovesiculating mutations faired poorly. The authors went on to argue that MVs serve as an export system to selectively eliminate unwanted material like misfolded proteins or regions of membrane compromised by interaction with polymyxin B. Other researchers have observed that MV release following exposure to antibiotics can contribute to the formation of biofilms. For example, He et al. (2019) reported that the treatment of methicillin-resistant *S. aureus* with β-lactam antibiotics induces biofilm formation. The ability of biofilms to limit the diffusion and efficacy of antibiotics is well established (Stewart, 2002).

In addition to short term resistance, we now have reason to believe that MVs promote the development of long-term adaptive resistance to antibiotics *via* the horizontal transfer of resistance genes. Recently, it was reported that glycine induced peptidoglycan defects in *E. coli* caused the bacteria to secrete MVs which carried plasmid DNA (Aktar et al., 2021). Though the peptidoglycan defects described in this paper arose from glycine, these findings beg the question: do antibiotics or other environmental influences which impact the peptidoglycan layer also favor the production of plasmid carrying MVs? Studies which document how changes which decrease peptidoglycan cross-linking increase the formation of membrane vesicles suggest this is a strong possibility (Wang et al., 2018). Consider this proposition in the context of a bacterial community which consists of various quasispecies in which some cells are carrying a resistance trait. When stressed by antibiotics, resistant bacteria upregulate the production of MVs which carry resistance plasmids. Subsequent MV uptake by other bacteria, even differing species, can then rapidly disseminate resistance traits across the community. Certainly, this story has been told for other mechanisms of horizontal gene transfer like transformation, transduction, and conjugation; however, the novelty of the MV packaging system is that it gives these antibiotic resistance elements a durable means to diffuse throughout a host and potentially persist into the greater environment.

The release of MVs in response to antibiotics may have more direct effects on host health as well. Recently, Kim et al. (2019) ran a series of *in vitro* experiments in which MVs were isolated from *E. faecium* grown under antibiotic stress. These MVs were then applied to Caco-2 cells. MVs produced by *E. faecium* exposed to linezolid were more cytotoxic toward Caco-2 cells than MVs from *E. faecium* grown in a non-antibiotic media. Similarly, MVs produced by *E. faecium* exposed to vancomycin stimulated more pro-inflammatory cytokine gene expression in Caco-2 cells (Kim et al., 2019). There is reason to believe that antibiotic generated MV effects might rise to the level of clinical significance.

Clinicians working in the field of infectious disease are familiar with the Jarisch-Herxheimer reaction in which the blood concentration of LPS endotoxins surges following the administration of antibiotics to patients infected with the phylum *Spirochaetes*. Interestingly, there is a delay between the lethal activity of the antibiotics and the release of endotoxin (Hurley, 1992). In view of our growing understanding of bacterial MVs, it is plausible that MVs may contribute to these or similar reactions. Less speculatively, recent work has demonstrated that imipenem increases infection related mortality to multi-drug resistant *Klebsiella pneumoniae* in a murine infection model (Ye et al., 2021). In this study, mice were infected with *Klebsiella* strains that were either susceptible or not susceptible to carbapenem antibiotics. Imipenem cured the infection of the antibiotic sensitive strain but increased the lethality of the carbapenem resistant strain. Further, imipenem also increased the levels of inflammatory cytokines IL-1β, IL-18, and TNF-α. The authors hypothesized that this observed increase in lethality resulted from mechanisms dependent on bacterial membrane vesicles. Previously, it has been shown that MVs from *Klebsiella* are phagocytized and that once internalized, these MVs interact with caspase-11, a pathogen associated molecular pattern (PAMP) receptor associated with phagocytotic cells (Vanaja et al., 2016). Caspase-11 activationin turn triggers the release of cytokine IL-1β and initiatespyroptosis (inflammatory cell death). In probing this hypothesis, they demonstrated that Imipenem enhances the release of MVs from *Klebsiella*. The enhanced lethality from imipenem could also be prevented through the administration of chemicals which inhibited the release of MVs; chemicals such as Cl-amidine hydrochloride, AMF30a, and GSK199.

## Vesicle Participation in Inter-Kingdom Signaling

**Figure 3:**
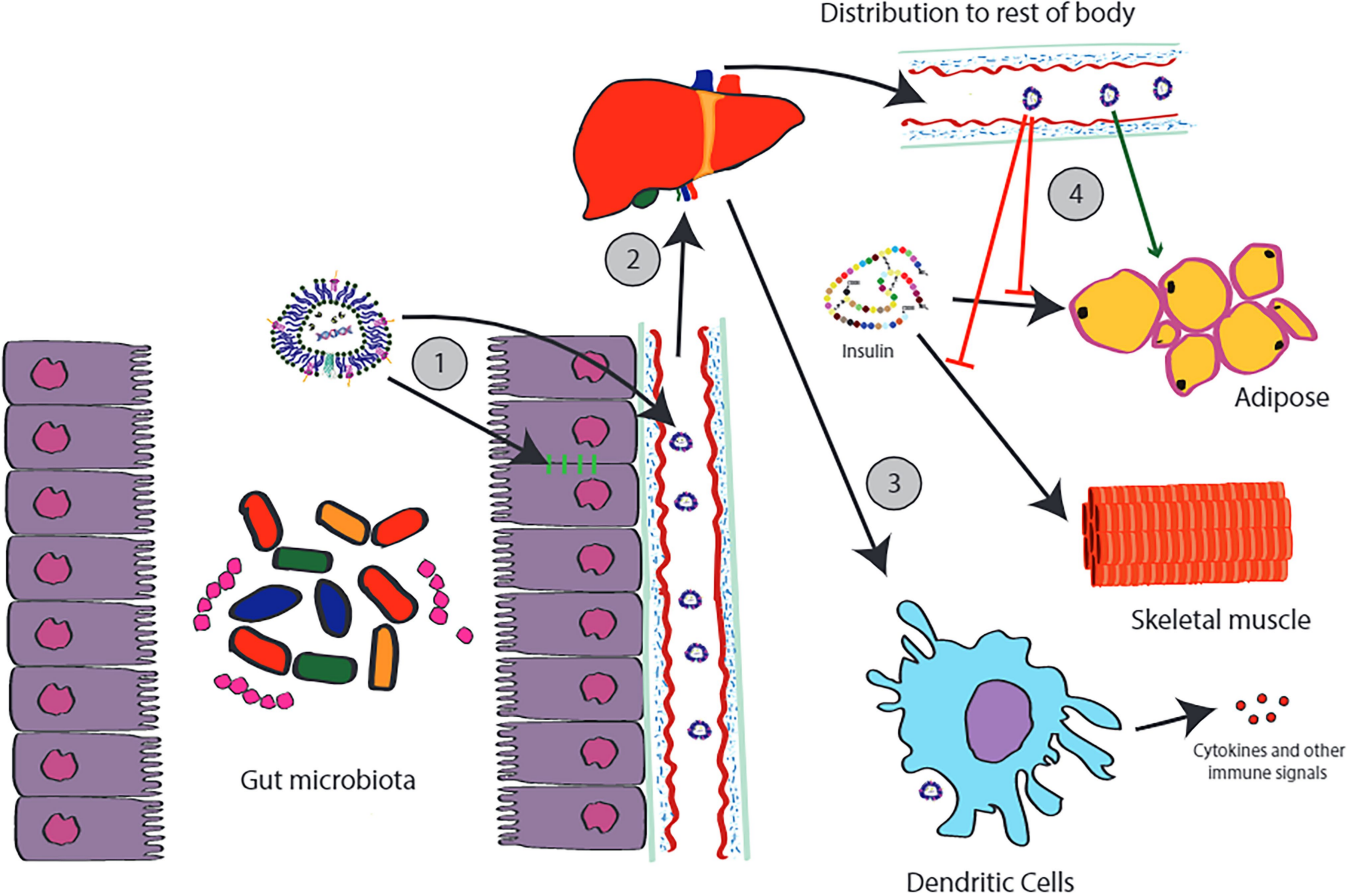
Examples of cross-kingdom signaling involving MVs produced by the gut microbiota. MVs produced by the gut microbiota can act locally, for example, increasing tight junction protein expression in the gut epithelial lining (1). Other MVs can transverse the gut lining where they enter the portal circulation. Most of the MVs that leave the gastrointestinal tract will accumulate in the liver (2). MVs accumulating in these tissues will interact with local dendritic cells, including the Kupffer cells of the liver. Depending on the composition of the MV, this can have a variety of effects. For example, MVs containing indole appear to be anti-inflammatory and appear to protect the liver against steatosis and injury. However, other types of MVs carry MAMPs which can trigger inflammatory responses from the immune system, such as the release of cytokines (3). Some MVs escape hepatic circulation and distribute throughout the rest of the body. Again, depending on the origin and composition of these MVs, differing effects may be observed. MVs from *Pseudomonas panacis* appear two interfere with the effects of insulin action on adipose tissue (4). In contrast, MVs from *Akkermansia muciniphilia* appeared to cause positive effects including a loss of body fat. This diagram is only a tiny portion of the full picture.

Much research to date has focused on peptides and the important roles these molecules facilitate in signaling. However, the time of the paradigm where peptides are viewed as the default signaling molecules has passed (Dauros et al., 2018). Recent discoveries have demonstrated host-microbe cross talk through small molecules including neurochemicals (Lyte, 2016), and signaling with molecules such as DNA and RNA (Knip et al., 2014; Koeppen et al., 2016; Lefebvre and Lécuyer, 2017). Given the ability for MVs to transport these sorts of signals (Figure 1), one wonders whether bacterial “signaling vesicles” exist and to what extent microbial MVs facilitate cross-communication with their host. We highlight some select examples of gut related MV cross-signaling in Figure 3.

There is evidence that MVs exert influence on the endocrine system and affect metabolism; findings that could prove highly relevant for diseases like type II diabetes. In a study conducted by Choi et al. (2015), MVs were isolated from the feces of high fat diet (HFD) and regular diet mice. These MVs were used to evaluate the potential for microbial MVs to influence the pathogenesis of type II diabetes. The authors found that MVs derived from HFD rodents were noticeably different from those of regular diet mice; HFD MV was smaller and demonstrated a distinct protein profile. More interestingly, MVs derived from HFD rodents interfered with insulin signaling and glucose uptake in skeletal muscle. Subsequent work identified these MV as products of the bacterium *Pseudomonas panacis*. When tested in isolation, MVs from *P. panacis* blocked the insulin signaling pathway in both skeletal muscle and adipose tissue. Moreover, when given orally, *P. panacis* MVs could induce diabetic phenotypes including glucose intolerance after glucose administration.

There is strong evidence to assert that bacterial MVs stimulate and influence the immune system. Later, we will discuss how this has served as the basis for novel MV based vaccines. However, aside from training the immune system, MVs laden with microbial products can more subtly modulate the immune system as well. In an experiment conducted by Li et al. (2004), MVs purified from the gram-positive bacteria *Lactobacillus rhamnosus* were tested in a series of *in vitro* assays and administered in a murine model of alcohol-associated liver disease (Gu et al., 2021). MVs from *L. rhamnosus* increased tight junction protein expression in epithelial cells and attenuated the LPS induced inflammatory response in macrophages. Additionally, these MVs also protected the liver against steatosis and injury. These protective effects were abolished in the presence of aryl-hydrocarbon receptor (AHR) inhibitors. The involvement of AHRs is highly relevant. AHRs recognize a variety of small molecules including the aromatic amine indole (Hubbard et al., 2015) and dopamine (Park et al., 2020). AHR receptors have garnered recent attention for their ability to modulate pathways influencing tissue homeostasis as well as pathological conditions ranging from inflammatory to neoplastic disorders (Rothhammer and Quintana, 2019). Indole derives primarily from the gut (Jaglin et al., 2018) and so does much peripheral dopamine (Eisenhofer et al., 1997).

Bacterial MVs have been found that contain DNA and RNA (Bitto et al., 2021). Bitto et al. (2021) purified MV from a multiple strains of *S. aureus* including methicillin resistant and sensitive clinical isolates. Following purification, MVs underwent DNA staining with SYTO-61 before visualization with confocal microscopy. DNA was detected, particularly at the surface of membrane vesicles. RNA content was also assessed, and samples contained RNA, predominantly sRNA of less than 100 nucleotides in length. Having detected the presence of both DNA and RNA, the researchers investigated whether these nucleic acid laden MVs could activate immune responses *via* pattern recognition receptors. They applied MVs to HEK-Blue reporter cells that expressed toll-like receptors including TLR2,4,7,8, and 9. Bacterial RNA is detected by TLR7 whereas bacterial DNA is detected by TLR 8 and 9. TLR7,8, and 9 were all activated in response to stimulation with increasing doses of MVs. The publication went on to show that MV stimulation induced pro-inflammatory cytokine responses including IL-6 (increases neutrophil production in bone marrow) and IL-8 (chemotaxis attractant for neutrophils). Interestingly, however, there were differences generated by different strains, which suggest differing proteomic profiles.

There are scattered other reports of MVs carrying sRNAs as well. The periodontal pathogens *Aggregatibacter actinomyce temcomitans*, *P. gingivalis*, and *Treponema denticola* have all been reported to produce miRNA-sized sRNAs (msRNAs) that are packed in MVs and have the capacity to induce the production of cytokines (Choi et al., 2017). It has also been shown that *P. aeruginosa*-derived methionine tRNA can be conveyed by MVs into human epithelial airway cells where it decreases the secretion of IL-8 (Koeppen et al., 2016). It is likely that with time we will see much more research in this area.

## A Potential Role for Vesicles in Health Maintenance

The effects of bacterial vesicles are not always detrimental. Earlier, we mentioned the work of Kwon et al. (2009) in which MVs derived from *P. panacis* induced diabetic effects. However, other members of the microbiota appear to make MVs with metabolically beneficial effects. In a study conducted by Ashrafian et al. (2019), MVs derived from *Akkermansia muciniphila* were given orally in a murine obesity model (Ashrafian et al., 2019). These MVs were able to effect changes in metabolism, causing a loss of body fat as well as an improvement in the lipid profile and glucose level of animals. There were also reported beneficial changes in intestinal barrier integrity and inflammation.

Because of their stability and ability to deliver select bacterial components in tandem with other immunogenic molecules, MVs are now being investigated as viable route to developing are providing vaccination. In a promising animal trial, orally administered MVs derived from *Vibrio cholera* were used to induce immunity against *V. cholera* O1 El Tor. The authors reported robust IgA and IgG responses which exceeded the response produced by a commercially available vaccine (Sedaghat et al., 2019). Reports of immunostimulatory MVs with therapeutic potential have also been reported for other species including *Streptococcus pneumoniae* (Olaya et al., 2014).

The ability to engineer MVs to transport specific cargo is of interest and MVs are being investigated as a platform to encapsulate and deliver small molecule drugs (Li and Liu, 2020). There are several advantages to this approach. MVs are thermodynamically stable and will not decompose in suspension. This is advantageous when trying to design drug preparations that need to have a longer shelf-life or demonstrate time-release kinetics. Further, a variety of molecules can be loaded into MVs. Small lipophilic molecules can often be loaded into MVs through simple passive diffusion whereas hydrophilic molecules can also be loaded into MVs using electroporation.

Bacterially expressed proteins can be selectively loaded into the MV of some species by inclusion of the OmpA signal peptide. Carvalho et al. (2019) reported modifying the Gram-negative organism *B. thetaiotaomicron* to secrete MVs that contained the human therapeutic protein, keratinocyte growth factor-2 (KGF-2). Purified MVs were tested in a murine, DSS-induced, acute colitis model. KGF-2 laden MVs demonstrated significant therapeutic potential by controlling colitis by both clinical and pathological measures. Among other notable effects, weight loss was reduced in animals receiving KGF-2 MVs, disease activity scores were lower for KGF-2 MVs treated groups, and histopathology showed that KGF-2 MV treatment reduced epithelial damage and inflammatory infiltration (Carvalho et al., 2019). Work is ongoing to create membrane vesicle vaccines for other bacteria including *S. aureus*. König et al. (2021) have described the development of an MV vaccine containing four *S. aureus* virulent factors, including ClfA_Y338A_, LukE, SpA_KKAA_, and Hla_H35L_ co-expressed in the same MV. The vaccine promoted opsonization and phagocytosis while limiting alpha-toxin mediated hemolysis, LukED-mediated leukocyte killing, and ClfA mediated binding of *S. aureus* to fibrinogen. In a murine model of infection, mice were robustly protected from *S. aureus* challenges. This promise of a MV based vaccine for humans recently became a reality: an MV based vaccine for *N. meningitidis* serogroup B received licensure and market authorization in Europe and the United States under the name Bexsero (Micoli and MacLennan, 2020). In the future, it is conceivable that engineering other types of proteins into bacterial MVs may allow for the development of vaccines targeted against non-bacterial pathogens such as viruses.

## Concluding Remarks and Future Perspectives

The importance of MVs in the pathogenesis of disease has been well-established by evidence from many laboratories. Exposure to environmental MVs can elicit potent immune reactions such as those responsible for allergic hypersensitivities. Interactions with the respiratory and integumentary systems are particularly relevant, but there is also evidence that ingested MVs can survive gastric passage and enter systemic circulation. Unfortunately, in our review of the literature, we did not find technical data detailing the effects of gastrointestinal conditions on MVs or much on consumed MVs in general. Further studies in this area could prove invaluable as the consumption of fermented products and probiotics is widespread.

We reviewed a variety of examples of microbes with MV cargos that are deleterious including the hydrolytic enzymes of *Pseudomonas* sp. or the porins of *N. gonorrhoeae*. In the case of microbes like *K. pneumoniae*, environmental insults such as antibiotics can trigger the release of MVs which exacerbate disease and propagate antibiotic resistance. These observations further the need for the responsible selection and application of antibiotics. While many studies have documented the relationship between antibiotic resistance and antibiotic misuse, very few studies have examined how changes in bacterial metabolism following exposure to antibiotics or other drugs can directly impact the host immune system or influence host-disease progression.

Membrane vesicle research conducted to date is largely concerned with the pathogenic potential of MVs. However, as work with lactic acid bacteria and select Gram-negatives like *Akkermansia muciniphilia* has shown, there is reason to believe that some exposure to MV might be beneficial. Further research is needed to assess the extent to which MVs might be involved in processes such as training the nascent immune system, downregulating inflammation, or in cross-kingdom signaling. The wider scientific community has only recently acknowledged the potential for Gram-positive microbes to generate MVs. Though it appears that MVs from Gram-positive microbes can carry cargos comparable to Gram-negative MV cargos, there also seem to be some unique differences between the two. Gram-positive microbes make a variety of unique metabolites that are worth investigating. The report in *L. rhamnosus* of MVs that appear to carry indole is of high interest for future work.

Other Gram-positive microbes have also been reported to produce indole and related immune-modulating molecules as well. Therefore, it is reasonable to assert that immune modulating MVs probably exist for many species. Consider the Gram-positive microbe *E. faecium*, which recently garnered attention for its ability to produce dopamine (Villageliú and Lyte, 2018; Rekdal et al., 2019). This finding holds implications for the management of Parkinson’s disease and is also being explored in a therapeutic context. Though dopamine is typically viewed from its role as a neurochemical, dopaminergic receptors exist throughout the immune system and dopamine serves as a bridging molecule between the immune and nervous systems (Sarkar et al., 2010). There is also evidence the microbe related dopamine is an important moderator of the host immune system and Zhang et al. convincingly demonstrated that microbe related peripheral (gut) dopamine suppresses iNKT cell-mediated hepatitis (Xue et al., 2018). Could findings like those reported by Zhang et al. be facilitated by the presence of a dopamine carrying MV? Recently, it has been learned that *E. faecium* produces MVs (Wagner et al., 2018; Kim et al., 2019) and now are we getting a glimpse into the composition of membrane vesicles in *E. faecium* and related species (Afonina et al., 2021). A dopamine bearing MV has not yet been described in the literature, but we offer this example to show how moving forward, it would be worth investigating the extent to which small molecular cargos like dopamine contribute to the composition of gram-positive MVs. Perhaps, a study of Gram-positive MVs will led to new examples of MVs which encapsulate beneficial microbial molecules. Moving into the future, the ability of MVs to encapsulate a variety of molecules also allows for the possibility for new drug and vaccine delivery systems.

## Author Contributions

The primary author, DV is a post-doctoral researcher in the lab of DS. DV reviewed the literature and prepared the manuscript under the guidance from DS who is an expert in the field of immunology. All authors contributed to the article and approved the submitted version.

## Funding

The work was supported by the National Institute on Alcohol Abuse and Alcoholism Grants: #K99-AA026336 and #R00-AA026336. The content is solely the responsibility of the authors and does not necessarily represent the official views of the National Institutes of Health. The funders had no role in study design, data collection and analysis, decision to publish, or preparation of manuscript.

## Conflict of Interest

The authors declare that the research was conducted in the absence of any commercial or financial relationships that could be construed as a potential conflict of interest.

## Publisher’s Note

All claims expressed in this article are solely those of the authors and do not necessarily represent those of their affiliated organizations, or those of the publisher, the editors and the reviewers. Any product that may be evaluated in this article, or claim that may be made by its manufacturer, is not guaranteed or endorsed by the publisher.

## References

[ref1] Abe K. Toyofuku M. Nomura N. Obana N. (2021). Autolysis-mediated membrane vesicle formation in *Bacillus subtilis*. Environ. Microbiol. 23, 2632–2647. doi: 10.1111/1462-2920.15502, PMID: 33817925

[ref2] Afonina I. Tien B. Nair Z. Matysik A. Lam L. N. Veleba M.. (2021). The composition and function of *enterococcus faecalis* membrane vesicles. microLife 2:uqab002. doi: 10.1093/femsml/uqab002 PMC10117786 37223255

[ref3] Aktar S. Okamoto Y. Ueno S. Tahara Y. O. Imaizumi M. Shintani M.. (2021). Incorporation of plasmid DNA into bacterial membrane vesicles by peptidogly can defects in *Escherichia coli*. Front. Microbiol. 12:747606. doi: 10.3389/fmicb.2021.747606, PMID: 34912309 PMC8667616

[ref4] Andreoni F. Toyofuku M. Menzi C. Kalawong R. Mairpady Shambat S. François P.. (2019). Antibiotics stimulate formation of vesicles in *Staphylococcus aureus* in both phage-dependent and -independent fashions and via different routes. Antimicrob. Agents Chemother. 63, e01439–e01418. doi: 10.1128/AAC.01439-18, PMID: 30509943 PMC6355553

[ref5] Ashrafian F. Shahriary A. Behrouzi A. Moradi H. R. Raftar S. K. A. Lari A.. (2019). Akkermansia muciniphila-derived extracellular vesicles as a mucosal delivery vector for amelioration of obesity in mice. Front. Microbiol. 10:2155. doi: 10.3389/fmicb.2019.02155, PMID: 31632356 PMC6779730

[ref6] Atilano M. L. Pereira P. M. Yates J. Reed P. Veiga H. Pinho M. G.. (2010). Teichoic acids are temporal and spatial regulators of peptidoglycan cross-linking in Staphylococcus aureus. Proc. Natl. Acad. Sci. 107, 18991–18996. doi: 10.1073/pnas.1004304107, PMID: 20944066 PMC2973906

[ref7] Bajic S. S. Cañas M.-A. Tolinacki M. Badia J. Sánchez B. Golic N.. (2020). Proteomic profile of extracellular vesicles released by *Lactiplantibacillus plantarum* BGAN8 and their internalization by non-polarized HT29 cell line. Sci. Rep. 10:21829. doi: 10.1038/s41598-020-78920-z, PMID: 33311536 PMC7732981

[ref8] Biller S. J. Lundeen R. A. Hmelo L. R. Becker K. W. Arellano A. A. Dooley K.. (2021). "Prochlorococcus extracellular vesicles: molecular composition and adsorption to diverse microbes." Environ. Microbiol. doi: 10.1111/1462-2920.15834 [Epub ahead of print]. PMC9298688 34766712

[ref9] Bitto N. J. Chapman R. Pidot S. Costin A. Lo C. Choi J.. (2017). Bacterial membrane vesicles transport their DNA cargo into host cells. Sci. Rep. 7:7072. doi: 10.1038/s41598-017-07288-4, PMID: 28765539 PMC5539193

[ref10] Bitto N. J. Cheng L. Johnston E. L. Pathirana R. Phan T. K. Poon I. K. H.. (2021). *Staphylococcus aureus* membrane vesicles contain immuno stimulatory DNA, RNA and peptidoglycan that activate innate immune receptors and induce autophagy. J. Extracell. Vesicles 10:e12080. doi: 10.1002/jev2.12080, PMID: 33815695 PMC8015888

[ref11] Bos J. Cisneros L. H. Mazel D. (2021). Real-time tracking of bacterial membrane vesicles reveals enhanced membrane traffic upon antibiotic exposure. Sci. Adv. 7:eabd1033. doi: 10.1126/sciadv.abd1033, PMID: 33523924 PMC7817102

[ref12] Briaud P. Frey A. Marino E. C. Bastock R. A. Zielinski R. E. Wiemels R. E.. (2021). Temperature influences the composition and cytotoxicity of extracellular vesicles in *Staphylococcus aureus*. mSphere 6:e0067621. doi: 10.1128/mSphere.00676-21, PMID: 34612674 PMC8510519

[ref13] Brown L. Wolf J. M. Prados-Rosales R. Casadevall A. (2015). Through the wall: extracellular vesicles in gram-positive bacteria, mycobacteria and fungi. Nat. Rev. Microbiol. 13, 620–630. doi: 10.1038/nrmicro3480, PMID: 26324094 PMC4860279

[ref14] Carvalho A. L. Fonseca S. Miquel-Clopés A. Cross K. Kok K.-S. Wegmann U.. (2019). Bioengineering commensal bacteria-derived outer membrane vesicles for delivery of biologics to the gastrointestinal and respiratory tract. J. Extracell. Vesicles 8:1632100. doi: 10.1080/20013078.2019.1632100, PMID: 31275534 PMC6598475

[ref15] Chattopadhyay M. K. Jaganandham M. V. (2015). Vesicles-mediated resistance to antibiotics in bacteria. Front. Microbiol. 6:758. doi: 10.3389/fmicb.2015.00758, PMID: 26257725 PMC4511839

[ref16] Chen A. Seifert H. S. (2014). Saturating mutagenesis of an essential gene: a majority of the *Neisseria gonorrhoeae* major outer membrane porin (PorB) is mutable. J. Bacteriol. 196, 540–547. doi: 10.1128/JB.01073-13, PMID: 24244002 PMC3911144

[ref17] Cheng A. G. McAdow M. Kim H. K. Bae T. Missiakas D. M. Schneewind O. (2010). Contribution of coagulases towards *Staphylococcus aureus* disease and protective immunity. PLoS Pathog. 6:e1001036. doi: 10.1371/journal.ppat.1001036, PMID: 20700445 PMC2916881

[ref18] Chia S. Qadan M. Newton R. Ludlam C. A. Fox K. A. Newby D. E. (2003). Intra-arterial tumor necrosis factor-alpha impairs endothelium-dependent vasodilatation and stimulates local tissue plasminogen activator release in humans. Arterioscler. Thromb. Vasc. Biol. 23, 695–701. doi: 10.1161/01.ATV.0000065195.22904.FA, PMID: 12692009

[ref19] Choi J. W. Kim S. C. Hong S. H. Lee H. J. (2017). Secretable small RNAs via outer membrane vesicles in periodontal pathogens. J. Dent. Res. 96, 458–466. doi: 10.1177/0022034516685071, PMID: 28068479

[ref20] Choi Y. Kwon Y. Kim D.-K. Jeon J. Jang S. C. Wang T.. (2015). Gut microbe-derived extracellular vesicles induce insulin resistance, thereby impairing glucose metabolism in skeletal muscle. Sci. Rep. 5:15878. doi: 10.1038/srep15878, PMID: 26510393 PMC4625370

[ref21] Cohen J. Abraham E. (1999). Microbiologic findings and correlations with serum tumor necrosis factor-alpha in patients with severe sepsis and septic shock. J. Infect. Dis. 180, 116–121. doi: 10.1086/314839, PMID: 10353869

[ref22] Dauros-Singorenko P. Blenkiron C. Phillips A. Swift S. (2018). The functional RNA cargo of bacterial membrane vesicles. FEMS Microbiol. Lett. 365:fny023. doi: 10.1093/femsle/fny023, PMID: 29390056

[ref23] Dean S. N. Leary D. H. Sullivan C. J. Oh E. Walper S. A. (2019). Isolation and characterization of *lactobacillus*-derived membrane vesicles. Sci. Rep. 9:877. doi: 10.1038/s41598-018-37120-6, PMID: 30696852 PMC6351534

[ref25] Deo P. Chow S. H. Han M.-L. Speir M. Huang C. Schittenhelm R. B.. (2020). Mitochondrial dysfunction caused by outer membrane vesicles from gram-negative bacteria activates intrinsic apoptosis and inflammation. Nat. Microbiol. 5, 1418–1427. doi: 10.1038/s41564-020-0773-2, PMID: 32807891

[ref26] Deo P. Chow S. H. Hay I. D. Kleifeld O. Costin A. Elgass K. D.. (2018). Outer membrane vesicles from *Neisseria gonorrhoeae* target PorB to mitochondria and induce apoptosis. PLoS Pathog. 14:e1006945. doi: 10.1371/journal.ppat.1006945, PMID: 29601598 PMC5877877

[ref27] Dorward D. W. Garon C. F. (1990). DNA is packaged within membrane-derived vesicles of gram-negative but not gram-positive bacteria. Appl. Environ. Microbiol. 56, 1960–1962. doi: 10.1128/aem.56.6.1960-1962.1990, PMID: 16348232 PMC184538

[ref28] Eisenhofer G. Aneman A. Friberg P. Hooper D. Fåndriks L. Lonroth H.. (1997). Substantial production of dopamine in the human gastrointestinal tract. J. Clin. Endocrinol. Metab. 82, 3864–3871. doi: 10.1210/jcem.82.11.4339, PMID: 9360553

[ref29] Ellis T. N. Kuehn M. J. (2010). Virulence and immunomodulatory roles of bacterial outer membrane vesicles. Microbiol. Mol. Biol. Rev. 74, 81–94. doi: 10.1128/MMBR.00031-09, PMID: 20197500 PMC2832350

[ref30] Ellis T. N. Leiman S. A. Kuehn M. J. (2010). Naturally produced outer membrane vesicles from *Pseudomonas aeruginosa* elicit a potent innate immune response via combined sensing of both lipopolysaccharide and protein components. Infect. Immun. 78, 3822–3831. doi: 10.1128/IAI.00433-10, PMID: 20605984 PMC2937433

[ref31] Fenster K. Freeburg B. Hollard C. Wong C. Rønhave Laursen R. Ouwehand A. C. (2019). The production and delivery of probiotics: a review of a practical approach. Microorganisms 7:83. doi: 10.3390/microorganisms7030083, PMID: 30884906 PMC6463069

[ref32] Fijan S. (2014). Microorganisms with claimed probiotic properties: an overview of recent literature. Int. J. Environ. Res. Public Health 11, 4745–4767. doi: 10.3390/ijerph110504745, PMID: 24859749 PMC4053917

[ref33] Gao L. van der Veen S. (2020). Role of outer membrane vesicles in bacterial physiology and host cell interactions. Infect. Microb. Dis. 2, 3–9. doi: 10.1097/IM9.0000000000000017, PMID: 31331976

[ref34] Gu Z. Li F. Liu Y. Jiang M. Zhang L. He L.. (2021). Exosome-like nanoparticles from *lactobacillus rhamnosus* GG protect against alcohol-associated liver disease through intestinal aryl hydrocarbon receptor in mice. Hepatol. Commun. 5, 846–864. doi: 10.1002/hep4.1679, PMID: 34027273 PMC8122379

[ref35] Hanson B. R. Neely M. N. (2012). Coordinate regulation of gram-positive cell surface components. Curr. Opin. Microbiol. 15, 204–210. doi: 10.1016/j.mib.2011.12.011, PMID: 22236805 PMC3320701

[ref36] Hanzelmann D. Joo H. S. Franz-Wachtel M. Hertlein T. Stevanovic S. Macek B.. (2016). Toll-like receptor 2 activation depends on lipopeptide shedding by bacterial surfactants. Nat. Commun. 7:12304. doi: 10.1038/ncomms12304, PMID: 27470911 PMC4974576

[ref37] He X. Li S. Yin Y. Xu J. Gong W. Li G.. (2019). Membrane vesicles are the dominant structural components of ceftazidime-induced biofilm formation in an oxacillin-sensitive MRSA. Front. Microbiol. 10:571. doi: 10.3389/fmicb.2019.00571, PMID: 30949156 PMC6438146

[ref38] Heyder J. (2004). Deposition of inhaled particles in the human respiratory tract and consequences for regional targeting in respiratory drug delivery. Proc. Am. Thorac. Soc. 1, 315–320. doi: 10.1513/pats.200409-046TA, PMID: 16113452

[ref39] Hong S. W. Kim M. R. Lee E. Y. Kim J. H. Kim Y. S. Jeon S. G.. (2011). Extracellular vesicles derived from *Staphylococcus aureus* induce atopic dermatitis-like skin inflammation. Allergy 66, 351–359. doi: 10.1111/j.1398-9995.2010.02483.x, PMID: 20831718 PMC3052535

[ref40] Horstman A. L. Kuehn M. J. (2000). Enterotoxigenic *Escherichia coli* secretes active heat-labile enterotoxin via outer membrane vesicles. J. Biol. Chem. 275, 12489–12496. doi: 10.1074/jbc.275.17.12489, PMID: 10777535 PMC4347834

[ref41] Hubbard T. D. Murray I. A. Bisson W. H. Lahoti T. S. Gowda K. Amin S. G.. (2015). Adaptation of the human aryl hydrocarbon receptor to sense microbiota-derived indoles. Sci. Rep. 5:12689. doi: 10.1038/srep12689, PMID: 26235394 PMC4522678

[ref42] Hurley J. C. (1992). Antibiotic-induced release of endotoxin: a reappraisal. Clin. Infect. Dis. 15, 840–854. doi: 10.1093/clind/15.5.840, PMID: 1445982

[ref43] Jaglin M. Rhimi M. Philippe C. Pons N. Bruneau A. Goustard B.. (2018). Indole, a signaling molecule produced by the gut microbiota, negatively impacts emotional behaviors in rats. Front. Neurosci. 12:216. doi: 10.3389/fnins.2018.00216, PMID: 29686603 PMC5900047

[ref44] Jiang Y. Kong Q. Roland K. L. Curtiss R. 3rd. (2014). Membrane vesicles of *Clostridium perfringens* type A strains induce innate and adaptive immunity. Int. J. Med. Microbiol. 304, 431–443. doi: 10.1016/j.ijmm.2014.02.006, PMID: 24631214 PMC4285460

[ref45] Jones E. J. Booth C. Fonseca S. Parker A. Cross K. Miquel-Clopés A.. (2020). The uptake, trafficking, and biodistribution of *Bacteroides thetaiotaomicron* generated outer membrane vesicles. Front. Microbiol. 11:57. doi: 10.3389/fmicb.2020.00057, PMID: 32117106 PMC7015872

[ref46] Jun S. H. Lee J. H. Kim B. R. Kim S. I. Park T. I. Lee J. C.. (2013). *Acinetobacter baumannii* outer membrane vesicles elicit a potent innate immune response via membrane proteins. PLoS One 8:e71751. doi: 10.1371/journal.pone.0071751, PMID: 23977136 PMC3743744

[ref47] Jung A. L. Stoiber C. Herkt C. E. Schulz C. Bertrams W. Schmeck B. (2016). *Legionella pneumophila*-derived outer membrane vesicles promote bacterial replication in macrophages. PLoS Pathog. 12:e1005592. doi: 10.1371/journal.ppat.1005592, PMID: 27105429 PMC4841580

[ref48] Kadurugamuwa J. L. Beveridge T. J. (1995). Virulence factors are released from *Pseudomonas aeruginosa* in association with membrane vesicles during normal growth and exposure to gentamicin: a novel mechanism of enzyme secretion. J. Bacteriol. 177, 3998–4008. doi: 10.1128/jb.177.14.3998-4008.1995, PMID: 7608073 PMC177130

[ref49] Kadurugamuwa J. L. Beveridge T. J. (1996). Bacteriolytic effect of membrane vesicles from *Pseudomonas aeruginosa* on other bacteria including pathogens: conceptually new antibiotics. J. Bacteriol. 178, 2767–2774. doi: 10.1128/jb.178.10.2767-2774.1996, PMID: 8631663 PMC178010

[ref50] Kadurugamuwa J. L. Beveridge T. J. (1997). Natural release of virulence factors in membrane vesicles by *Pseudomonas aeruginosa* and the effect of aminoglycoside antibiotics on their release. J. Antimicrob. Chemother. 40, 615–621. doi: 10.1093/jac/40.5.615, PMID: 9421308

[ref51] Kim S. H. Kim K. S. Lee S. R. Kim E. Kim M. S. Lee E. Y.. (2009). Structural modifications of outer membrane vesicles to refine them as vaccine delivery vehicles. Biochim. Biophys. Acta 1788, 2150–2159. doi: 10.1016/j.bbamem.2009.08.001, PMID: 19695218 PMC5007125

[ref52] Kim M. H. Kim S. Y. Son J. H. Kim S. I. Lee H. Kim S.. (2019). Production of membrane vesicles by *enterococcus faecium* cultured with or without subinhibitory concentrations of antibiotics and their pathological effects on epithelial cells. Front. Cell. Infect. Microbiol. 9:295. doi: 10.3389/fcimb.2019.00295, PMID: 31475120 PMC6702262

[ref53] Kim J. H. Lee J. Park J. Gho Y. S. (2015). Gram-negative and gram-positive bacterial extracellular vesicles. Semin. Cell Dev. Biol. 40, 97–104. doi: 10.1016/j.semcdb.2015.02.006, PMID: 25704309

[ref54] Klieve A. Yokoyama M. Forster R. Ouwerkerk D. Bain P. Mawhinney E. (2005). Naturally occurring DNA transfer system associated with membrane vesicles in cellulolytic *Ruminococcus* spp. of ruminal origin. Appl. Environ. Microbiol. 71, 4248–4253. doi: 10.1128/AEM.71.8.4248-4253.2005, PMID: 16085810 PMC1183309

[ref55] Knip M. Constantin M. E. Thordal-Christensen H. (2014). Trans-kingdom cross-talk: small RNAs on the move. PLoS Genet. 10:e1004602. doi: 10.1371/journal.pgen.1004602, PMID: 25188222 PMC4154666

[ref56] Koeppen K. Hampton T. H. Jarek M. Scharfe M. Gerber S. A. Mielcarz D. W.. (2016). A novel mechanism of host-pathogen interaction through sRNA in bacterial outer membrane vesicles. PLoS Pathog. 12:e1005672. doi: 10.1371/journal.ppat.1005672, PMID: 27295279 PMC4905634

[ref57] Kolling G. L. Matthews K. R. (1999). Export of virulence genes and Shiga toxin by membrane vesicles of *Escherichia coli* O157:H7. Appl. Environ. Microbiol. 65, 1843–1848. doi: 10.1128/AEM.65.5.1843-1848.1999, PMID: 10223967 PMC91264

[ref58] König E. Gagliardi A. Riedmiller I. Andretta C. Tomasi M. Irene C.. (2021). Multi-antigen outer membrane vesicle engineering to develop polyvalent vaccines: the *Staphylococcus aureus* case. Front. Immunol. 12:752168. doi: 10.3389/fimmu.2021.752168, PMID: 34819933 PMC8606680

[ref59] Konings W. N. Bisschop A. Veenhuis M. Vermeulen C. A. (1973). New procedure for the isolation of membrane vesicles of *Bacillus subtilis* and an electron microscopy study of their ultrastructure. J. Bacteriol. 116, 1456–1465. doi: 10.1128/jb.116.3.1456-1465.1973, PMID: 4201775 PMC246505

[ref60] Kozjak-Pavlovic V. Dian-Lothrop E. A. Meinecke M. Kepp O. Ross K. Rajalingam K.. (2009). Bacterial porin disrupts mitochondrial membrane potential and sensitizes host cells to apoptosis. PLoS Pathog. 5:e1000629. doi: 10.1371/journal.ppat.1000629, PMID: 19851451 PMC2759283

[ref61] Kuehn M. J. Kesty N. C. (2005). Bacterial outer membrane vesicles and the host-pathogen interaction. Genes Dev. 19, 2645–2655. doi: 10.1101/gad.1299905, PMID: 16291643

[ref62] Kulp A. Kuehn M. J. (2010). Biological functions and biogenesis of secreted bacterial outer membrane vesicles. Annu. Rev. Microbiol. 64, 163–184. doi: 10.1146/annurev.micro.091208.073413, PMID: 20825345 PMC3525469

[ref63] Kwon S.-O. Gho Y. S. Lee J. C. Kim S. I. (2009). Proteome analysis of outer membrane vesicles from a clinical *Acinetobacter baumannii* isolate. FEMS Microbiol. Lett. 297, 150–156. doi: 10.1111/j.1574-6968.2009.01669.x, PMID: 19548894

[ref64] Lähteenmäki K. Kuusela P. Korhonen T. K. (2001). Bacterial plasminogen activators and receptors. FEMS Microbiol. Rev. 25, 531–552. doi: 10.1016/S0168-6445(01)00067-5, PMID: 11742690

[ref65] Lee J. (2012). *Staphylococcus aureus* membrane vesicles and its potential role in bacterial pathogenesis. J. Bacteriol. Virol. 42:181. doi: 10.4167/jbv.2012.42.3.181, PMID: 34623928

[ref66] Lee E.-Y. Bang J. Y. Park G. W. Choi D.-S. Kang J. S. Kim H.-J.. (2007). Global proteomic profiling of native outer membrane vesicles derived from *Escherichia coli*. Proteomics 7, 3143–3153. doi: 10.1002/pmic.200700196, PMID: 17787032

[ref67] Lee E. Y. Choi D. Y. Kim D. K. Kim J. W. Park J. O. Kim S.. (2009). Gram-positive bacteria produce membrane vesicles: proteomics-based characterization of *Staphylococcus aureus*-derived membrane vesicles. Proteomics 9, 5425–5436. doi: 10.1002/pmic.200900338, PMID: 19834908

[ref68] Lee H. J. Hong S. H. (2012). Analysis of microRNA-size, small RNAs in *Streptococcus mutans* by deep sequencing. FEMS Microbiol. Lett. 326, 131–136. doi: 10.1111/j.1574-6968.2011.02441.x, PMID: 22092283

[ref69] Lee J. Lee E.-Y. Kim S.-H. Kim D.-K. Park K.-S. Kim K. P.. (2013). *Staphylococcus aureus* extracellular vesicles carry biologically activ;-lactamase. Antimicrob. Agents Chemother. 57, 2589–2595. doi: 10.1128/AAC.00522-12, PMID: 23529736 PMC3716153

[ref70] Lefebvre F. A. Lécuyer E. (2017). Small luggage for a long journey: transfer of vesicle-enclosed small RNA in interspecies communication. Front. Microbiol. 8:377. doi: 10.3389/fmicb.2017.00377, PMID: 28360889 PMC5352665

[ref71] Lendak D. F. Mihajlović D. M. Novakov-Mikić A. S. Mitić I. M. Boban J. M. Brkić S. V. (2018). The role of TNF-α superfamily members in immunopathogenesis of sepsis. Cytokine 111, 125–130. doi: 10.1016/j.cyto.2018.08.015, PMID: 30142533

[ref72] Li R. Liu Q. (2020). Engineered bacterial outer membrane vesicles as multifunctional delivery platforms. Front. Mater. 7:202. doi: 10.3389/fmats.2020.00202

[ref73] Li Z. S. Pham T. D. Tamir H. Chen J. J. Gershon M. D. (2004). Enteric dopaminergic neurons: definition, developmental lineage, and effects of extrinsic denervation. J. Neurosci. 24, 1330–1339. doi: 10.1523/JNEUROSCI.3982-03.2004, PMID: 14960604 PMC6730344

[ref74] Lyte M. (2016). Microbial endocrinology: an ongoing personal journey. Adv. Exp. Med. Biol. 874, 1–24. doi: 10.1007/978-3-319-20215-0_1, PMID: 26589212

[ref75] Macdonald I. A. Kuehn M. J. (2013). Stress-induced outer membrane vesicle production by *Pseudomonas aeruginosa*. J. Bacteriol. 195, 2971–2981. doi: 10.1128/JB.02267-12, PMID: 23625841 PMC3697536

[ref76] Manning A. J. Kuehn M. J. (2011). Contribution of bacterial outer membrane vesicles to innate bacterial defense. BMC Microbiol. 11:258. doi: 10.1186/1471-2180-11-258, PMID: 22133164 PMC3248377

[ref77] Marsollier L. Brodin P. Jackson M. Korduláková J. Tafelmeyer P. Carbonnelle E.. (2007). Impact of *mycobacterium ulcerans* biofilm on transmissibility to ecological niches and Buruli ulcer pathogenesis. PLoS Pathog. 3:e62. doi: 10.1371/journal.ppat.0030062, PMID: 17480118 PMC1864991

[ref78] McBroom A. J. Kuehn M. J. (2007). Release of outer membrane vesicles by gram-negative bacteria is a novel envelope stress response. Mol. Microbiol. 63, 545–558. doi: 10.1111/j.1365-2958.2006.05522.x, PMID: 17163978 PMC1868505

[ref79] McMahon K. J. Castelli M. E. García Vescovi E. Feldman M. F. (2012). Biogenesis of outer membrane vesicles in Serratia marcescens is thermoregulated and can be induced by activation of the Rcs phosphorelay system. J. Bacteriol. 194, 3241–3249. doi: 10.1128/JB.00016-12, PMID: 22493021 PMC3370869

[ref80] Meganathan V. Moyana R. Natarajan K. Kujur W. Kusampudi S. Mulik S.. (2020). Bacterial extracellular vesicles isolated from organic dust induce neutrophilic inflammation in the lung. Am. J. Phys. Lung Cell. Mol. Phys. 319, L893–L907. doi: 10.1152/ajplung.00107.2020, PMID: 32996778 PMC7792685

[ref81] Mendez J. A. Soares N. C. Mateos J. Gayoso C. Rumbo C. Aranda J.. (2012). Extracellular proteome of a highly invasive multidrug-resistant clinical strain of *Acinetobacter baumannii*. J. Proteome Res. 11, 5678–5694. doi: 10.1021/pr300496c, PMID: 22966805

[ref82] Micoli F. MacLennan C. A. (2020). Outer membrane vesicle vaccines. Semin. Immunol. 50:101433. doi: 10.1016/j.smim.2020.101433, PMID: 33309166

[ref83] Mitaka C. Hirata Y. Ichikawa K. Yokoyama K. Emori T. Kanno K.. (1994). Effects of TNF-alpha on hemodynamic changes and circulating endothelium-derived vasoactive factors in dogs. Am. J. Phys. 267, H1530–H1536. doi: 10.1152/ajpheart.1994.267.4.H1530, PMID: 7943398

[ref84] Mozaheb N. Mingeot-Leclercq M.-P. (2020). Membrane vesicle production as a bacterial defense against stress. Front. Microbiol. 11:600221. doi: 10.3389/fmicb.2020.600221, PMID: 33362747 PMC7755613

[ref85] Nagakubo T. Nomura N. Toyofuku M. (2020). Cracking open bacterial membrane vesicles. Front. Microbiol. 10:3026. doi: 10.3389/fmicb.2019.03026, PMID: 32038523 PMC6988826

[ref86] Ñahui Palomino R. A. Vanpouille C. Costantini P. E. Margolis L. (2021). Microbiota-host communications: bacterial extracellular vesicles as a common language. PLoS Pathog. 17:e1009508. doi: 10.1371/journal.ppat.1009508, PMID: 33984071 PMC8118305

[ref87] Nevermann J. Silva A. Otero C. Oyarzún D. P. Barrera B. Gil F.. (2019). Identification of genes involved in biogenesis of outer membrane vesicles (OMVs) in *salmonella enterica* serovar typhi. Front. Microbiol. 10:104. doi: 10.3389/fmicb.2019.00104, PMID: 30778340 PMC6369716

[ref88] Nishiyama K. Takaki T. Sugiyama M. Fukuda I. Aiso M. Mukai T.. (2020). Extracellular vesicles produced by *Bifidobacterium longum* export mucin-binding proteins. Appl. Environ. Microbiol. 86, e01464–e01420. doi: 10.1128/AEM.01464-20, PMID: 32737132 PMC7499026

[ref89] O'Donoghue E. J. Krachler A. M. (2016). Mechanisms of outer membrane vesicle entry into host cells. Cell. Microbiol. 18, 1508–1517. doi: 10.1111/cmi.12655, PMID: 27529760 PMC5091637

[ref90] Olaya-Abril A. Prados-Rosales R. McConnell M. J. Martín-Peña R. González-Reyes J. A. Jiménez-Munguía I.. (2014). Characterization of protective extracellular membrane-derived vesicles produced by *Streptococcus pneumoniae*. J. Proteome 106, 46–60. doi: 10.1016/j.jprot.2014.04.023, PMID: 24769240

[ref92] Park H. Jin U. H. Karki K. Jayaraman A. Allred C. Michelhaugh S. K.. (2020). Dopamine is an aryl hydrocarbon receptor agonist. Biochem. J. 477, 3899–3910. doi: 10.1042/BCJ20200440, PMID: 32905582 PMC7772691

[ref93] Prados-Rosales R. Weinrick B. C. Piqué D. G. Jacobs W. R. Jr. Casadevall A. Rodriguez G. M. (2014). Role for *mycobacterium tuberculosis* membrane vesicles in iron acquisition. J. Bacteriol. 196, 1250–1256. doi: 10.1128/JB.01090-13, PMID: 24415729 PMC3957709

[ref94] Rekdal V. M. Bess E. N. Bisanz J. E. Turnbaugh P. J. Balskus E. P. (2019). Discovery and inhibition of an interspecies gut bacterial pathway for levodopa metabolism. Science 364:eaau6323. doi: 10.1126/science.aau6323, PMID: 31196984 PMC7745125

[ref950] Rivera J. Cordero R. J. B. Nakouzi A. S. Frases A. Nicola P. J. Casadevall A. (2010). Bacillus anthracis produces membrane-derived vesicles containing biologically active toxins. Proceedings of the National Academy of Sciences 107, 19002–19007., PMID: 20956325 10.1073/pnas.1008843107 PMC2973860

[ref95] Roden J. A. Wells D. H. Chomel B. B. Kasten R. W. Koehler J. E. (2012). Hemin binding protein C is found in outer membrane vesicles and protects *Bartonella henselae* against toxic concentrations of hemin. Infect. Immun. 80, 929–942. doi: 10.1128/IAI.05769-11, PMID: 22232189 PMC3294634

[ref97] Rothhammer V. Quintana F. J. (2019). The aryl hydrocarbon receptor: an environmental sensor integrating immune responses in health and disease. Nat. Rev. Immunol. 19, 184–197. doi: 10.1038/s41577-019-0125-8, PMID: 30718831

[ref98] Rueter C. Bielaszewska M. (2020). Secretion and delivery of intestinal pathogenic *Escherichia coli* virulence factors via outer membrane vesicles. Front. Cell. Infect. Microbiol. 10:91. doi: 10.3389/fcimb.2020.00091, PMID: 32211344 PMC7068151

[ref99] Sabnis A. Ledger E. V. K. Pader V. Edwards A. M. (2018). Antibiotic interceptors: creating safe spaces for bacteria. PLoS Pathog. 14:e1006924. doi: 10.1371/journal.ppat.1006924, PMID: 29672612 PMC5908065

[ref100] Salvachúa D. Werner A. Z. Pardo I. Michalska M. Black B. A. Donohoe B. S.. (2020). Outer membrane vesicles catabolize lignin-derived aromatic compounds in *pseudomonas* putidaKT2440. Proc. Natl. Acad. Sci. 117, 9302–9310. doi: 10.1073/pnas.1921073117, PMID: 32245809 PMC7196908

[ref101] Sarkar C. Basu B. Chakroborty D. Dasgupta P. S. Basu S. (2010). The immunoregulatory role of dopamine: an update. Brain Behav. Immun. 24, 525–528. doi: 10.1016/j.bbi.2009.10.015, PMID: 19896530 PMC2856781

[ref102] Schlatterer K. Beck C. Hanzelmann D. Lebtig M. Fehrenbacher B. Schaller M.. (2018). The mechanism behind bacterial lipoprotein release: phenol-soluble modulins mediate toll-like receptor 2 activation via extracellular vesicle release from *Staphylococcus aureus*. MBio 9, e01851–e01818. doi: 10.1128/mBio.01851-18, PMID: 30459192 PMC6247081

[ref103] Sedaghat M. Siadat S. D. Mirabzadeh E. Keramati M. Vaziri F. Shafiei M.. (2019). Evaluation of antibody responses to outer membrane vesicles (OMVs) and killed whole cell of vibrio cholerae O1 El tor in immunized mice. Iranian J. Microbiol. 11, 212–219. PMID: 31523404 PMC6711870

[ref105] Spoerry C. Seele J. Valentin-Weigand P. Baums C. G. von Pawel-Rammingen U. (2016). Identification and characterization of IgdE, a novel IgG-degrading protease of *Streptococcus suis* with unique specificity for porcine IgG. J. Biol. Chem. 291, 7915–7925. doi: 10.1074/jbc.M115.711440, PMID: 26861873 PMC4824999

[ref106] Stentz R. Horn N. Cross K. Salt L. Brearley C. Livermore D. M.. (2015). Cephalosporinases associated with outer membrane vesicles released by *Bacteroides* spp. protect gut pathogens and commensals against β-lactam antibiotics. J. Antimicrob. Chemother. 70, 701–709. doi: 10.1093/jac/dku466, PMID: 25433011 PMC4319488

[ref107] Stewart P. S. (2002). Mechanisms of antibiotic resistance in bacterial biofilms. Int. J. Med. Microbiol. 292, 107–113. doi: 10.1078/1438-4221-00196, PMID: 12195733

[ref108] Tamang J. P. Cotter P. D. Endo A. Han N. S. Kort R. Liu S. Q.. (2020). Fermented foods in a global age: east meets west. Compr. Rev. Food Sci. Food Saf. 19, 184–217. doi: 10.1111/1541-4337.12520, PMID: 33319517

[ref109] Thomas R. J. (2013). Particle size and pathogenicity in the respiratory tract. Virulence 4, 847–858. doi: 10.4161/viru.27172, PMID: 24225380 PMC3925716

[ref110] Toledo A. Coleman J. L. Kuhlow C. J. Crowley J. T. Benach J. L. (2012). The enolase of *Borrelia burgdorferi* is a plasminogen receptor released in outer membrane vesicles. Infect. Immun. 80, 359–368. doi: 10.1128/IAI.05836-11, PMID: 22083700 PMC3255694

[ref111] Toyofuku M. Nomura N. Eberl L. (2019). Types and origins of bacterial membrane vesicles. Nat. Rev. Microbiol. 17, 13–24. doi: 10.1038/s41579-018-0112-2, PMID: 30397270

[ref112] Turnbull L. Toyofuku M. Hynen A. L. Kurosawa M. Pessi G. Petty N. K.. (2016). Explosive cell lysis as a mechanism for the biogenesis of bacterial membrane vesicles and biofilms. Nat. Commun. 7:11220. doi: 10.1038/ncomms11220, PMID: 27075392 PMC4834629

[ref113] Vanaja S. K. Russo A. J. Behl B. Banerjee I. Yankova M. Deshmukh S. D.. (2016). Bacterial outer membrane vesicles mediate cytosolic localization of LPS and caspase-11 activation. Cell 165, 1106–1119. doi: 10.1016/j.cell.2016.04.015, PMID: 27156449 PMC4874922

[ref114] Villageliú D. Lyte M. (2018). Dopamine production in *enterococcus faecium*: a microbial endocrinology-based mechanism for the selection of probiotics based on neurochemical-producing potential. PLoS One 13:e0207038. doi: 10.1371/journal.pone.0207038, PMID: 30485295 PMC6261559

[ref115] Vincent C. D. Friedman J. R. Jeong K. C. Buford E. C. Miller J. L. Vogel J. P. (2006). Identification of the core transmembrane complex of the *legionella* dot/Icm type IV secretion system. Mol. Microbiol. 62, 1278–1291. doi: 10.1111/j.1365-2958.2006.05446.x, PMID: 17040490

[ref116] Vitse J. Devreese B. (2020). The contribution of membrane vesicles to bacterial pathogenicity in cystic fibrosis infections and healthcare associated pneumonia. Front. Microbiol. 11:630. doi: 10.3389/fmicb.2020.00630, PMID: 32328052 PMC7160670

[ref117] Wagner T. Joshi B. Janice J. Askarian F. Škalko-Basnet N. Hagestad O. C.. (2018). *Enterococcus faecium* produces membrane vesicles containing virulence factors and antimicrobial resistance related proteins. J. Proteome 187, 28–38. doi: 10.1016/j.jprot.2018.05.017, PMID: 29857065

[ref118] Wang X. Koffi P. F. English O. F. Lee J. C. (2021). *Staphylococcus aureus* extracellular vesicles: a story of toxicity and the stress of 2020. Toxins 13:75. doi: 10.3390/toxins13020075, PMID: 33498438 PMC7909408

[ref119] Wang X. Thompson C. D. Weidenmaier C. Lee J. C. (2018). Release of *Staphylococcus aureus* extracellular vesicles and their application as a vaccine platform. Nat. Commun. 9:1379. doi: 10.1038/s41467-018-03847-z, PMID: 29643357 PMC5895597

[ref120] Wawrzeniak K. Gaur G. Sapi E. Senejani A. G. (2020). Effect of *Borrelia burgdorferi* outer membrane vesicles on host oxidative stress response. Antibiotics 9:275. doi: 10.3390/antibiotics9050275, PMID: 32466166 PMC7277464

[ref121] Xue R. Zhang H. Pan J. Du Z. Zhou W. Zhang Z.. (2018). Peripheral dopamine controlled by gut microbes inhibits invariant natural killer T cell-mediated hepatitis. Front. Immunol. 9:2398. doi: 10.3389/fimmu.2018.02398, PMID: 30386344 PMC6199378

[ref122] Yang J. Kim Y. K. Kang T. S. Jee Y. K. Kim Y. Y. (2017). Importance of indoor dust biological ultrafine particles in the pathogenesis of chronic inflammatory lung diseases. Environ. Health Toxicol. 32:e2017021. doi: 10.5620/eht.e2017021, PMID: 29161804 PMC5735549

[ref123] Yang J. Kim E. K. Park H. J. McDowell A. Kim Y.-K. (2020). The impact of bacteria-derived ultrafine dust particles on pulmonary diseases. Exp. Mol. Med. 52, 338–347. doi: 10.1038/s12276-019-0367-3, PMID: 32203101 PMC7156658

[ref124] Ye C. Li W. Yang Y. Liu Q. Li S. Zheng P.. (2021). Inappropriate use of antibiotics exacerbates inflammation through OMV-induced pyroptosis in MDR *Klebsiella pneumoniae* infection. Cell Rep. 36:109750. doi: 10.1016/j.celrep.2021.109750, PMID: 34551309

[ref125] Yokoyama K. Horii T. Yamashino T. Hashikawa S. Barua S. Hasegawa T.. (2000). Production of Shiga toxin by *Escherichia coli* measured with reference to the membrane vesicle-associated toxins. FEMS Microbiol. Lett. 192, 139–144. doi: 10.1111/j.1574-6968.2000.tb09372.x, PMID: 11040442

[ref126] Yonezawa H. Osaki T. Woo T. Kurata S. Zaman C. Hojo F.. (2011). Analysis of outer membrane vesicle protein involved in biofilm formation of *Helicobacter pylori*. Anaerobe 17, 388–390. doi: 10.1016/j.anaerobe.2011.03.020, PMID: 21515394

[ref127] Yu Y.-J. Wang X.-H. Fan G.-C. (2018). Versatile effects of bacterium-released membrane vesicles on mammalian cells and infectious/inflammatory diseases. Acta Pharmacol. Sin. 39, 514–533. doi: 10.1038/aps.2017.82, PMID: 28858295 PMC5888691

